# Overexpression of Nanog in amniotic fluid–derived mesenchymal stem cells accelerates dermal papilla cell activity and promotes hair follicle regeneration

**DOI:** 10.1038/s12276-019-0266-7

**Published:** 2019-07-04

**Authors:** Junghyun Park, Eun Kyoung Jun, Daryeon Son, Wonjun Hong, Jihoon Jang, Wonjin Yun, Byung Sun Yoon, Gwonhwa Song, In Yong Kim, Seungkwon You

**Affiliations:** 1 Department of Biotechnology, College of Life Sciences and Biotechnology, Korea University, Seoul, 02841 South Korea; 2 Institute of Regenerative Medicine, STEMLAB, Inc., Seoul, 02841 Republic of Korea; 3 Department of Neurosurgery, College of Medicine, Korea University, Seoul, 02841 South Korea; 4 Institute of Animal Molecular Biotechnology, Korea University, Seoul, 136-701 South Korea

**Keywords:** Mesenchymal stem cells, Regeneration, Self-renewal

## Abstract

Alopecia, one of the most common chronic diseases, can seriously affect a patient’s psychosocial life. Dermal papilla (DP) cells serve as essential signaling centers in the regulation of hair growth and regeneration and are associated with crosstalk between autocrine/paracrine factors and the surrounding environment. We previously demonstrated that amniotic fluid–derived mesenchymal stem cell–conditioned medium (AF-MSC-CM) accelerates hair regeneration and growth. The present study describes the effects of overexpression of a reprogramming factor, Nanog, on MSC properties, the paracrine effects on DP cells, and in vivo hair regrowth. First, we examined the in vitro proliferation and lifespan of AF-MSCs overexpressing reprogramming factors, including Oct4, Nanog, and Lin28, alone or in combination. Among these factors, Nanog was identified as a key factor in maintaining the self-renewal capability of AF-MSCs by delaying cellular senescence, increasing the endogenous expression of Oct4 and Sox2, and preserving stemness. Next, we evaluated the paracrine effects of AF-MSCs overexpressing Nanog (AF-N-MSCs) by monitoring secretory molecules related to hair regeneration and growth (IGF, PDGF, bFGF, and Wnt7a) and proliferation of DP cells. In vivo studies revealed that CM derived from AF-N-MSCs (AF-N-CM) accelerated the telogen-to-anagen transition in hair follicles (HFs) and increased HF density. The expression of DP and HF stem cell markers and genes related to hair induction were higher in AF-N-CM than in CM from AF-MSCs (AF-CM). This study suggests that the secretome from autologous MSCs overexpressing Nanog could be an excellent candidate as a powerful anagen inducer and hair growth stimulator for the treatment of alopecia.

## Alopecia: Stimulating hair growth via stem cell-based therapy

A treatment developed using stem cells that express high levels of a hair-growth-stimulating protein could help treat alopecia. Cells at the base of hair follicles secrete signaling molecules that help maintain follicle stem cell populations and stimulate hair growth, but defects in these signaling pathways can trigger alopecia. Potential treatments created using stem cells derived from amniotic fluid are being explored by Gwonhwa Song, In Yong Kim and Seungkwon You at the College of Life Sciences and Biotechnology, Seoul, South Korea. They have demonstrated that a protein called Nanog found in amniotic fluid-derived stem cell populations can help maintain stem cell health and stimulate secretion of hair growth proteins. These findings led them to develop a topical treatment with medicines derived from stem cells overexpressing Nanog, which triggered dermal cell proliferation and hair regrowth in mice.

## Introduction

Alopecia, the general medical term for hair loss or baldness, is a distressing condition caused by complex factors, including genetic, hormonal, iatrogenic, and traumatic events. Although alopecia is not a life-threatening disease, it is associated with a variety of negative psychosocial impacts in sufferers. In particular, androgenetic alopecia, which is characterized by progressive miniaturization of hair follicles (HFs), occurs in 50% of menopausal women, 35% of women of childbearing age, and 70% of adult men^1^. Despite its high prevalence, the approved therapeutic options are limited to several oral and topical agents, and patients’ expectations are much higher than the reality^2^. Hair transplantation is the second-best option for alopecia patients who do not have success with medical therapies; however, previous studies reported conflicting results due to differences in techniques, skill levels of the surgeons who performed the transplant, and characteristics of the patients. Moreover, such operations cannot be performed more than three times on the same patient^3^. Accordingly, increased attention has been paid to developing satisfactory first-line treatments.

The HF is a self-renewing mini-organ containing dermal and epidermal compartments that undergoes a cyclic process of growth, regression, and resting phases (anagen, catagen, and telogen) over the lifetime of the organism to continuously produce new hair shafts^4^. The dermal papilla (DP) is a key signaling center responsible for the maintenance of hair-inducing activity, as well as the regeneration and development of HFs^5^. The powerful androgen dihydrotestosterone (DHT) inhibits proliferation of HaCaT keratinocytes in coculture with DP cells obtained from androgenetic alopecia patients by suppressing Wnt signal–mediated transcription^6^. In addition, another study using outer root sheath keratinocytes in a coculture system revealed that DHT-inducible dickkopf 1 expressed in DP cells leads to apoptosis of the follicular cells^7^. These results are reminiscent of the phenotype observed in androgenetic alopecia patients, in whom the HF cycle is interrupted during the telogen phase, and terminal scalp hairs are replaced by smaller hairs. Similarly, when a patient experiences a hormonal imbalance in androgenic hormones, such as testosterone, DP cells undergo apoptosis, pausing the cycle of hair growth and ultimately resulting in hair loss^8^. Furthermore, androgen-driven alterations in autocrine or paracrine factors secreted from DP cells are closely associated with defects in HF stem cell function^9^. Therefore, improved proliferative homeostasis and maintenance of the HF stem cell population would be an important step toward the development of new treatments for alopecia. Meanwhile, recent studies have shown that mesenchymal stem cells (MSCs) produce paracrine molecules, allowing DP cells to promote hair growth^10,11^. Similarly, we showed previously that conditioned medium (CM) derived from human amniotic fluid (AF)-MSCs accelerates wound healing, thereby promoting hair regeneration^12^. Notwithstanding the paracrine effect of MSCs on hair growth, more evidence of efficacy is still required before clinical applications. In addition, understanding the MSC-derived paracrine signals responsible for the DP functions would provide a pathway to more efficacious, personalized, and targeted treatment options.

Nanog plays a pivotal role in preventing differentiation to primitive endoderm and maintaining pluripotency of embryonic stem cells (ESCs)^13^. Consistent with this, downregulation of pluripotency-related genes, such as Oct4, Sox2, and Nanog, induces ESC differentiation into extraembryonic lineages^14^. In contrast, elevated Nanog expression in ESCs contributes to the activation of pluripotency genes and yields of hybrid colonies when these ESCs fuse to differentiated cells^15^. More recently, the pluripotency-related properties of Nanog have been applied to cell reprogramming to generate induced pluripotent stem cells or cause metabolic changes. For instance, Tsai et al. reported that overexpression of Nanog and Oct4 in MSCs allows cells to maintain long-term self-renewal and stemness in an undifferentiated state^16^.

In this study, we first examined how overexpression of pluripotency-related transcription factors affects proliferation and lifespan of AF-MSCs and preservation of their MSC properties. To investigate Nanog-induced cytokine changes in MSCs, we compared the secretory molecules derived from normal AF-MSCs and Nanog-overexpressing AF-MSCs (AF-N-MSCs) and then evaluated the paracrine effects of Nanog-overexpressing MSCs on DP cell proliferation. Finally, we prepared CM from AF-N-MSCs (AF-N-CM) and assessed its effects on in vivo hair regrowth.

## Materials and methods

### Culture of AF-MSCs and CM preparation

The AF-MSCs used in the present study were approved by and used with strict adherence to the guidelines of the Institutional Review Board of Korea University, Seoul, Korea (KUGH16060-001). AF-MSCs were obtained by amniocentesis performed for fetal karyotyping between 16 and 20 weeks of gestation. AF-MSCs, previously characterized as MSCs by verification of their differentiation, proliferation, and immunological phenotypes^17^, were used in this study. All experiments were performed in triplicate on independently isolated AF-MSCs. For overexpression of pluripotent transcription factors, AF-MSCs were cultured in low-glucose Dulbecco’s modified Eagle’s medium (DMEM), 10% fetal bovine serum (FBS), 100 U penicillin/streptomycin, 1% L-glutamine, 4 ng/mL basic fibroblast growth factor (bFGF; R&D Systems, Minneapolis, MN, USA), 5 ng/mL sodium selenite (Sigma-Aldrich, S9133, St. Louis, MO, USA), and 50 μg/mL ascorbic acid (proliferation media). To prepare AF-MSC-CM, AF-MSCs were plated at a concentration of 5 × 10^5^ cells/100 mm plate and incubated in proliferation medium. After attaching, the AF-MSCs were switched to serum-free high-glucose DMEM and incubated for 3 more days. The resultant medium was collected, centrifuged at 500 × *g* for 5 min, and filtered through a 0.20-μm syringe filter as previously described^12^; the filtrate was used in subsequent experiments as CM.

### Overexpression of pluripotency-related transcription factors

The coding sequences for human Nanog genes were amplified by reverse transcriptase–polymerase chain reaction (RT-PCR) with Phusion High-Fidelity polymerase (New England Biolab, Ipswich, MA, USA) and primers specific for Nanog (5′-ACTATTTAAACTCGAGCCACCATGAGTGTGGATCCAGCTTGTCC-3′ and 5′- CTGGCGGCCGCTCGATCACACGTCTTCAGGTTGCATGT-3′). The amplified fragments were cloned into the NotI-digested pMXs vector (Cell Biolabs). pMXs vectors containing Oct4 (#17217) and Lin28 (#47902) genes were purchased from Addgene (Watertown, MA, USA). Recombinant vectors were transfected into 293 GPG packaging cells using Lipofectamine 2000 (Thermo Fisher Scientific, Waltham, MA, USA) according to the manufacturer’s protocol. The viruses were collected after 24 h and filtered through a 0.45-μm filter before transduction. For AF-MSC infection, harvested viruses were added to proliferation media and incubated with the cells for 6 h. After washing with phosphate-buffered saline (PBS), the cells were incubated in proliferation media.

### AF-MSC proliferation rate assay

To measure the growth of AF-MSCs, the cells were cultured in triplicate at a density of 3 × 10^4^ cells/well (in 12-well plates) in growth medium for 3 days, stained with 0.01% crystal violet solution, destained with 10% acetic acid, and subjected to spectrophotometric analysis (absorbance at 600 nm) to determine relative cell growth rates.

### Colony-forming unit fibroblast (CFU-F) assay

AF-MSCs and AF-N-MSCs were seeded in 6-well plates at a density of 100 cells per well, cultured for 14 days, washed twice with PBS, and fixed in 10% formalin for 20 min at room temperature. To visualize colonies, cells were stained with 0.01% crystal violet solution for 20 min at room temperature, washed with deionized water, and air-dried. Colonies were scored macroscopically.

### Extraction of RNA and transcriptome microarrays

Total RNA extracted from AF and AF-N samples was prepared for microarrays. The quality and quantity of the extracted RNA were examined by an RNA quantification reagent (Ribogreen®, Invitrogen, Carlsbad, CA, USA). Fluorescence was determined using a fluorometer (Victor 3, Perkin Elmer, Boston, MA, USA). RNA was converted to cDNA, amplified, and purified using an Illumina Total Prep RNA Amplification Kit (Ambion, Carlsbad CA, USA). The HumanHT-12 BeadChip contained sequences representing approximately 47,315 probes, which represent 27,455 curated and putative genes. An Illumina iScan scanner was used to create images of the microarrays. The intensities of the images were measured using GenomeStudio (v.2011.1, Illumina, Inc., San Diego, CA, USA) with the Gene Expression (v.1.0) module software. The expression value of each gene was determined by calculating differences by perfect match intensity minus mismatch intensity of the probe pairs in use.

### Adipogenic differentiation

Differentiation of MSCs was performed as previously described^17^. Briefly, 3 × 10^4^ MSCs/well were seeded in 6-well culture dishes and cultured in proliferation media to reach 100% confluence. The cells were then sequentially cultured in adipogenic induction medium [high-glucose DMEM (Invitrogen, Carlsbad, CA, USA) supplemented with 33 μM biotin, 17 μM pantothenate, 10 mM acetic acid, 5 mM dexamethasone, 0.5 mM 3-isobutyl-1-methyl-xanthine (Sigma-Aldrich), 10 ng/mL recombinant human insulin (Sigma-Aldrich), and 10% FBS] for 7 days. This process was repeated three times. After the final differentiation, the cells were fixed with 10% formalin (Sigma-Aldrich), washed with PBS, and stained with 2% (w/v) Oil Red O (Sigma-Aldrich) for 20 min at room temperature to detect oil droplets in the cytoplasm.

### Osteogenic differentiation

Differentiation of MSCs was performed as previously described^17^. Briefly, 3 × 10^4^ cells/well were seeded in 6-well culture dishes, cultured in proliferation media to 70–80% confluence, and then cultured for 2.5 weeks in osteogenic induction medium [Iscove’s Modified Dulbecco’s Medium (IMDM) basal medium (Gibco/Invitrogen) supplemented with 5 μM dexamethasone, 10 mM β-glycerophosphate, 0.2 mM ascorbate, and 10% FBS] or control medium (IMDM basal medium supplemented with 10% FBS). Osteogenic differentiation was confirmed by fixing cells with 10% formalin (Sigma-Aldrich) for 15 min at room temperature and staining with silver nitrate.

### Chondrogenic differentiation

Differentiation of MSCs was performed as previously described^17^. Briefly, cells were detached from culture dishes, transferred into a 15-mL polypropylene tube, centrifuged at 1000 rpm for 5 min to form a pelleted micromass at the bottom of the tube, and then treated with chondrogenic medium [high-glucose DMEM supplemented with 0.1 M dexamethasone, 50 g/mL AsA (Sigma-Aldrich), 100 g/mL sodium pyruvate (Sigma-Aldrich), 40 g/mL proline (Sigma-Aldrich), 10 ng/mL transforming growth factor-1 (R&D Systems, Minneapolis, MN, USA), and 50 mg/mL ITSpremix (Gibco/Invitrogen), 6.25 µg/mL insulin, 6.25 µg/mL transferrin (Sigma-Aldrich), 6.25 ng/mL selenious acid (Sigma-Aldrich), 1.25 mg/mL bovine serum albumin, and 5.35 mg/mL linoleic acid (Sigma-Aldrich)] and control medium (high-glucose DMEM). Medium changes were carried out twice weekly, and chondrogenesis was assessed at weekly intervals. After 4 weeks of culture, the cells were washed twice with PBS, fixed in 4% paraformaldehyde, and visualized by staining with Alcian Blue (Sigma-Aldrich).

### RT-PCR and quantitative real-time PCR

RNA from MSCs was prepared using TRIzol according to the manufacturer’s instructions (Invitrogen), and cDNA was synthesized using Reverse Transcriptase II (RT, Invitrogen). To amplify specific nucleic acid sequences, 25 ng of cDNA was combined with the corresponding pair of PCR primers (Bioneer, Daejeon, Korea). The PCR program consisted of 24–30 cycles of the following process: melting at 94 °C for 30 s, annealing at 62 °C for 30 s, and extension at 72 °C for 30 s, followed by a final amplification step for 10 min at 72 °C. The linear range of target markers was confirmed by electrophoresis. Real-time RT-PCR was performed on an iCycler IQ (Bio-Rad, Hercules, CA, USA) with SYBR Green PCR Master Mix (Bio-Rad). mRNA levels were normalized against the expression levels of glyceraldehyde-3-phosphatedehydrogenase in parallel with those of the target marker genes. Primer sequences are listed in Supplemental Table 1. Fold change was calculated by the ΔΔCt method^18^.

### Western blotting

To detect overexpressed transcription factors and secreted cytokines in AF-MSCs, 3 × 10^5^ cells were plated on 100 mm plates and cultured in proliferation media for 48 h. In the case of tissue samples for specific hair regrowth markers, the obtained tissues were ground using a homogenizer. Total protein was extracted with RIPA buffer, and the mixture was centrifuged at 12,000 × *g* for 30 min at 4 °C. For normalization of relative expression, protein concentrations were measured using the Bradford Assay Kit (Bio-Rad). Extracted proteins were separated on precast 4–12% gradient sodium dodecyl sulfate-polyacrylamide gel electrophoresis (Invitrogen) by electrophoresis and then transferred onto polyvinylidene difluoride membranes (Millipore, Bedford, MA, USA). For immunological reactions, membranes were incubated with the indicated primary antibodies at 4 °C overnight. Primary antibodies are listed in Supplemental Table 2. Blots were incubated with secondary antibodies (horseradish peroxidase-conjugated anti-mouse or anti-rabbit IgG) for 1 h at room temperature and then imaged using an ECL Kit (Pierce, Rockford, IL, USA) on an ImageQuant LAS 4000 imager (GE Healthcare Life Sciences, Chicago, IL, USA).

### Reactive oxygen species (ROS) analysis

DHE (Invitrogen), an oxidative fluorescent dye (red color), was used to detect superoxide (O_2_^−^). Briefly, cells were seeded in 6-well plates and allowed to attach for approximately 8 h. Subsequently, the cells were treated with 10 μM DHE for 30 min at 37 °C in an incubator protected from light. The DHE-treated cells were washed with PBS and fixed with 4% formalin in PBS.

### Fluorescence-activated cell sorting (FACS) analysis

AF-MSCs were trypsinized and transferred into FACS tubes at 1 × 10^6^ cells/tube (BD Biosciences Clontech, Palo Alto, CA, USA). After two rinses with cold PBS, the cells were incubated at 37 °C for 30 min with 10 μM DHE. After incubation, the cells were washed twice with PBS, fixed with a fixative solution (0.5% paraformaldehyde in PBS), and then subjected to FACS analysis.

### Enzyme-linked immunosorbent assay (ELISA)

Levels of paracrine factors in CM (AF-CM and AF-N-CM) were measured by ELISA (RayBiotech Inc., Norcross, GA, USA). To measure the levels of cytokines in CM, 100 µL of prepared standards and samples were added to a precoated strip microplate. After overnight incubation at 4 °C, 100 µL of biotin antibody was added to each well and incubated for 1 h at room temperature. Streptavidin solution (100 µL) was added to each sample and incubated for 45 min at RT. Next, TMB 1-Step Solution (Thermo Fisher, Waltham, MA, USA) was added to detect chemiluminescence, which was detected at 450 nm. Delta values were normalized based on the extinction of the standard curves, and protein content was calculated for each condition.

### In vitro DP proliferation assay

Human HFDP cells (CB-HDP-001) were purchased from Cell Engineering for Origin (Seoul, Korea). For growth of HFDP cells after each treatment, HFDP were cultured at a density of 3 × 10^4^ cells/well (in 12-well plates) in triplicate in AF-CM, AF-N-CM, and 1 μM minoxidil for 2 and 4 days and then stained with 0.01% crystal violet solution. The crystal violet solution was extracted using 10% acetic acid and measured by spectrophotometry (absorbance at 600 nm) to determine the relative cell growth rates.

### In vivo hair regeneration assay

All animal procedures were approved by the Institutional Animal Care & Use Committee of Korea University (KUIACUC-2017–75). C57BL/6 mice (6 weeks old; female; body weight, 20 g) were obtained from Dae Han Bio Link (Chungcheongbuk-do, Korean). Mice were randomly divided into three groups, and the hair regeneration model was generated. The second anagen stage was induced by plucking the dorsal skin of mice in the telogen phase of the hair cycle. After hair removal, CM (AF, AF-N, or minoxidil) was topically applied to the region in a volume of 50 µL. A quick-bonding adhesive (Tegaderm, 3M, St. Paul, MN, USA) was used to fix the splint to the skin and CM, thereby stabilizing its position over the dorsal region. Digital photographs of the dorsal region were taken at 0, 5, and 10 days. For histological analysis, samples of dorsal skin were harvested. The dorsal skins were fixed with 4% paraformaldehyde and processed by paraffin block embedding. The general HF tissue was visualized by hematoxylin–eosin staining and observed by microscopy (Olympus DP70, Tokyo, Japan). AP staining was performed according to the manufacturer’s instructions (Vector Laboratories, Burlingame, CA, USA). Frozen sections (8-µm thick) were treated with 100 mM Tris-HCl (pH 8.2) containing 0.1% Tween. Working solution (combination of reagents 1–3) was added to skin samples and incubated for 20 min at RT. After washing with PBS, the AP-stained tissue was observed and quantified by ImageJ. For immunofluorescence of HFs, dorsal skins were harvested and fixed in 4% paraformaldehyde. Fixed samples were embedded in OCT at −20 °C overnight. The frozen sections (8-µm thick) were incubated with anti-mouse CK15 antibody (Thermo Fisher, Waltham, MA, USA) at 4 °C overnight. After washing with PBS, the samples were incubated with Cy3-labeled secondary antibodies (1:500) for 2 h and washed twice with PBS. Stained sections were observed and imaged on an Olympus DP70 camera system (Olympus America, Inc., Waltham, MA, USA).

### Statistical analysis

All values are expressed as the mean ± SD. Data comparisons were performed using one- or two-way analyses of variance with post hoc Tukey’s test and paired two-tailed Student’s *t* tests. Differences with *p* < 0.001, *p* < 0.01, and *p* < 0.05 were considered statistically significant.

## Results

### Forced expression of pluripotency-related transcription factors for promoting stemness in AF-MSCs

**Fig. 1:**
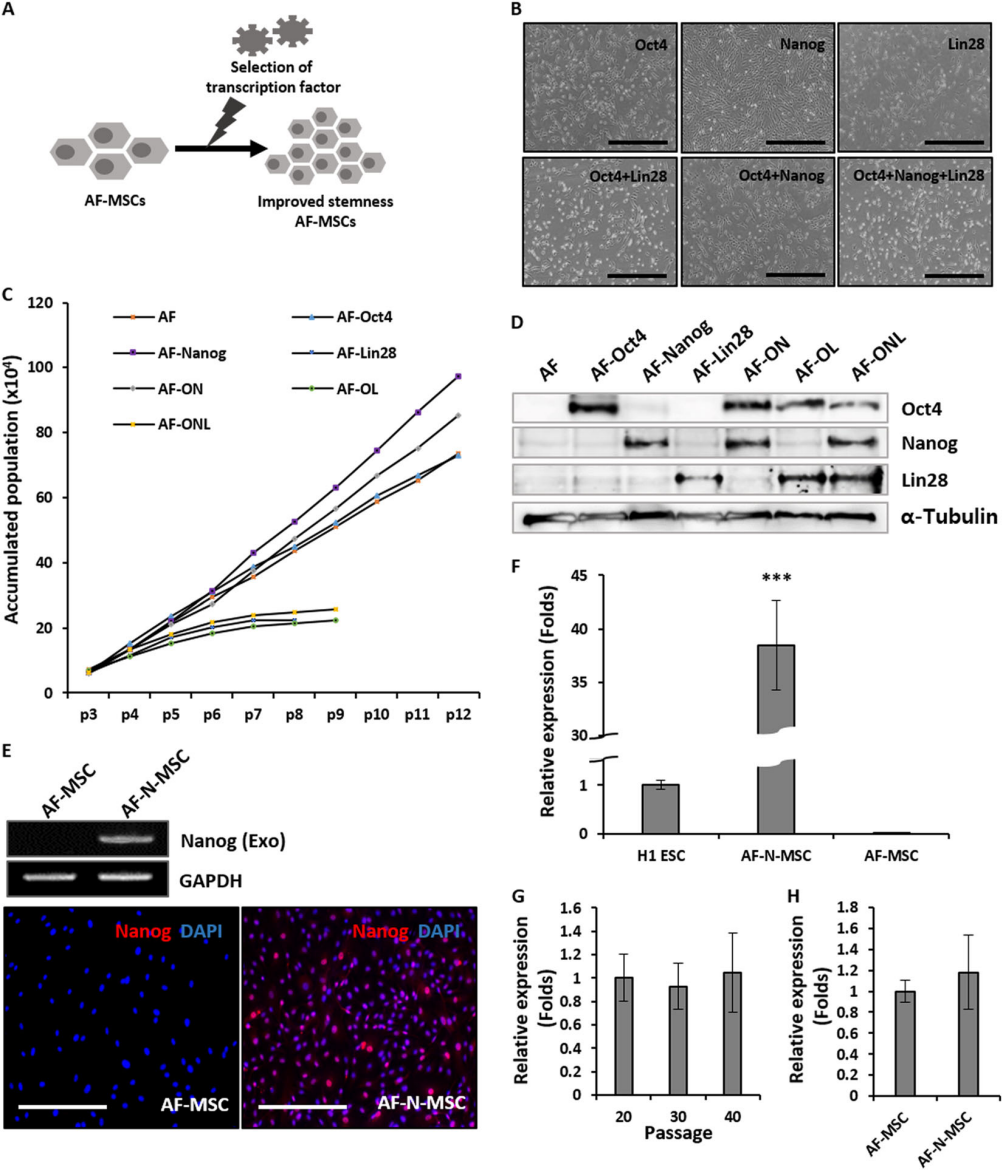
Forced expression of pluripotency-related transcription factors in amniotic fluid–derived mesenchymal stem cells (AF-MSCs). **a** Schematic of the process for selecting a key transcription factor to promote stemness of AF-MSCs. **b** Morphological changes and **c** growth rate of AF-MSCs infected with retroviral vectors encoding Oct4, Nanog, and Lin28, alone or in combination. Scale bar, 1 mm. **d** Expression of transcription factors, alone or in combination, as determined by western blotting. **e** Overexpression of exogenous Nanog in AF-MSCs, detected by reverse transcriptase–polymerase chain reaction (RT-PCR) and immunofluorescence. Scale bar, 1 mm. **f** Nanog expression in H1 embryonic stem cells, AF-MSCs overexpressing Nanog (AF-N-MSCs), and AF-MSCs. **g** Expression of Nanog in AF-N (infected at 10 passages) over 40 passages. **h** Expression of endogenous Nanog in AF-N-MSCs, detected by quantitative real-time RT-PCR. Data are represented as the mean ± SD (*n* = 3). ***p < 0.001

We hypothesized that overexpression of pluripotency-related transcription factors would contribute to the stem cell properties of MSCs (Fig. 1a). To test this hypothesis, we infected AF-MSCs with retroviral vectors encoding Oct4, Nanog, and Lin28, either alone or in combination (Fig. 1b). Figure 1b, c shows the morphological changes of the AF-MSCs at 6 days post-infection and their growth rates for 12 passages in culture. Expression of exogenous transcription factors in all cells was confirmed by western blot analyses (Fig. 1d). Forced expression of Oct4, with or without other factors, led to limited self-renewal and morphological changes, and overexpression of Lin28 dramatically decreased cell growth and induced cell senescence. The forced expression of Nanog alone exhibited the greatest proliferative potential. Interestingly, a significant difference was found in the growth rates of AF-MSCs depending on the participation of Lin28. Based on these results, we suggest that Nanog is the key transcriptional regulator responsible for the enhanced proliferation of AF-MSCs.

**Fig. 2:**
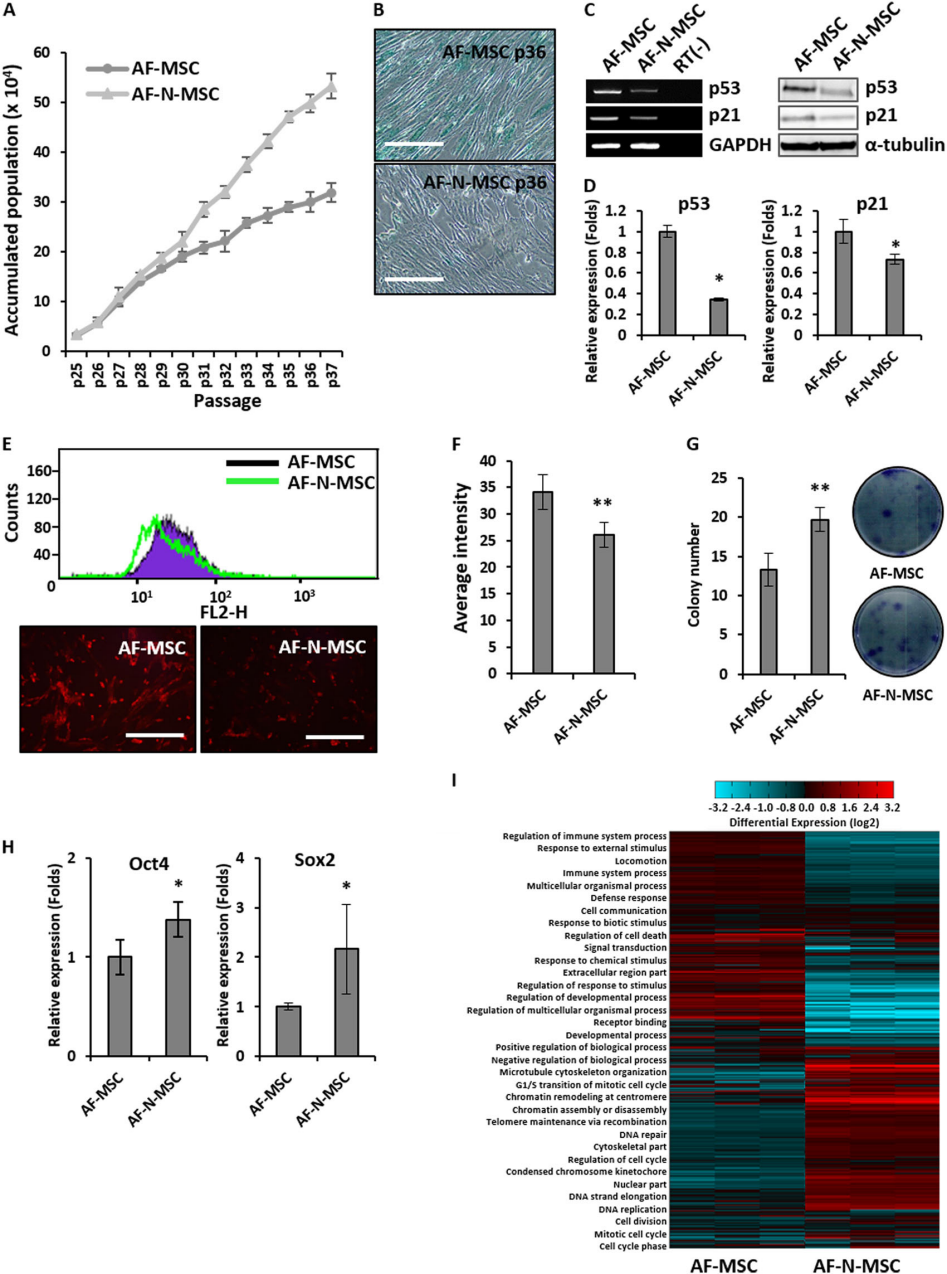
Promotion of amniotic fluid–derived mesenchymal stem cell (AF-MSC) stemness by Nanog overexpression. **a** Growth rate of AF-MSCs overexpressing Nanog (AF-N-MSCs). Non-modified AF-MSCs served as a control (AF-MSCs). **b** β-Gal staining of AF- and AF-N-MSCs in passage 36. Scale bar, 200 µm. **c** mRNA (left) and protein expression (right) of senescence-related genes in AF- and AF-N-MSCs. **d** Quantification of p53 and p21 expression by quantitative real-time reverse transcriptase–polymerase chain reaction (qRT-PCR). **e** Fluorescence-activated cell sorting analysis and DHE staining to detect reactive oxygen species (ROS) accumulation in AF- and AF-N-MSCs. Scale bar, 1 mm. **f** Quantification of fluorescence intensity for ROS accumulation. **g** Colony-forming unit fibroblast analysis for evaluation of AF- and AF-N-MSC stemness. **h** Changes in endogenous expression of the pluripotency-related transcription factors (Oct4 and Sox2) in AF-MSCs upon Nanog overexpression. Expression levels were measured by RT-PCR and qRT-PCR. **i** Hierarchical clustering analysis of gene expression in AF- and AF-N-MSCs. Data are represented as the mean ± SD (*n* = 3). *p < 0.05; **p < 0.01

To further characterize the roles of Nanog, we generated AF-N-MSCs from three different AF samples and compared their proliferative capacities using non-modified AF-MSCs as a control. Whereas the expression of Nanog was almost undetectable in unmodified AF-MSCs, its forced expression could be confirmed by RT-PCR and immunostaining; the overexpressed protein was localized exclusively in the nucleus (Fig. 1e). To clarify the overexpression of Nanog in AF-N-MSCs, we compared its expression in ESCs (referred to as H1 ESCs) and unmodified AF-MSCs. The results showed that the Nanog expression level in AF-N-MSCs was approximately 40-fold higher than in H1 ESCs and 1600-fold greater than in AF-MSCs (Fig. 1f). The expression of Nanog in AF-N-MSCs was continued over 40 passages (Fig. 1g). Endogenous Nanog expression was barely influenced by overexpression of Nanog (Fig. 1h). As shown in Fig. 2a, the accumulated populations of both AF-MSCs and AF-N-MSCs increased over 37 passages, although AF-N-MSCs eventually proliferated significantly faster. SA-β-gal staining performed at passage 36 revealed that Nanog delayed cellular senescence (Fig. 2b). The expression of p53 and p21 mRNA and proteins at passage 32 was lower in AF-N-MSCs than in AF-MSCs, potentially explaining the reduced growth arrest of AF-N-MSCs (Fig. 2c, d). Because intracellular accumulation of ROS accelerates passage-dependent senescence of MSCs^19^, we compared ROS levels in AF- and AF-N-MSCs by DHE staining at passage 32. A significant difference was observed in the ROS levels depending on the presence or absence of Nanog amplification (Fig. 2e, f). Taken together, these findings indicate that overexpression of Nanog delays replicative senescence in human AF-MSCs, thereby promoting their proliferation.

A CFU-F assay was performed to investigate the effect of Nanog overexpression on the stemness of AF-MSCs. The results showed that the number of colonies in AF-N-MSCs was approximately 1.5-fold higher than in AF-MSCs (Fig. 2g). Furthermore, since pluripotency genes are known to regulate the stemness of MSCs and ESCs by maintaining self-renewal and inhibiting differentiation^20,21^, we investigated the endogenous expression of Oct4 and Sox2 in AF-N-MSCs to determine whether the enrichment of Nanog protein has a profound effect on the expression of other pluripotency factors. The endogenous expression of Oct4 and Sox2 was higher in AF-N-MSCs than in AF-MSCs (Fig. 2h). Meanwhile, we compared the biological processes and pathways of AF-N-MSCs with AF-MSCs through 5216 genes (Fig. 2i). We confirmed that significantly upregulated genes (1865) and significantly downregulated genes (1850) were detected in AF-N-MSCs compared to AF-MSCs. Compared to AF-MSCs, the gene ontology categories that were more significantly enriched in AF-N-MSCs were gene sets related to the regulation of the cell cycle (SKP2, CyclinA2, CyclinE2, etc.), DNA replication (PRIM1, CDC25A, LIG1, etc.), cell division (CDCAs, KIFs, RCC1, etc.), and chromatin remodeling (CHD7, OIP5, etc.); meanwhile, the genes related to immune system processes (CXCLs, TLR3, TNF, etc.), cell death (CCLs, ICAM1, etc.), response to stimulus (VCAM1, TCF21, ITGA10, etc.), and signal transduction (GPR4, LCP1, SIK1, etc.) were highly downregulated in AF-N-MSCs. These results clearly showed a higher expression of proliferation-related genes in AF-N-MSCs following Nanog overexpression.

**Fig. 3:**
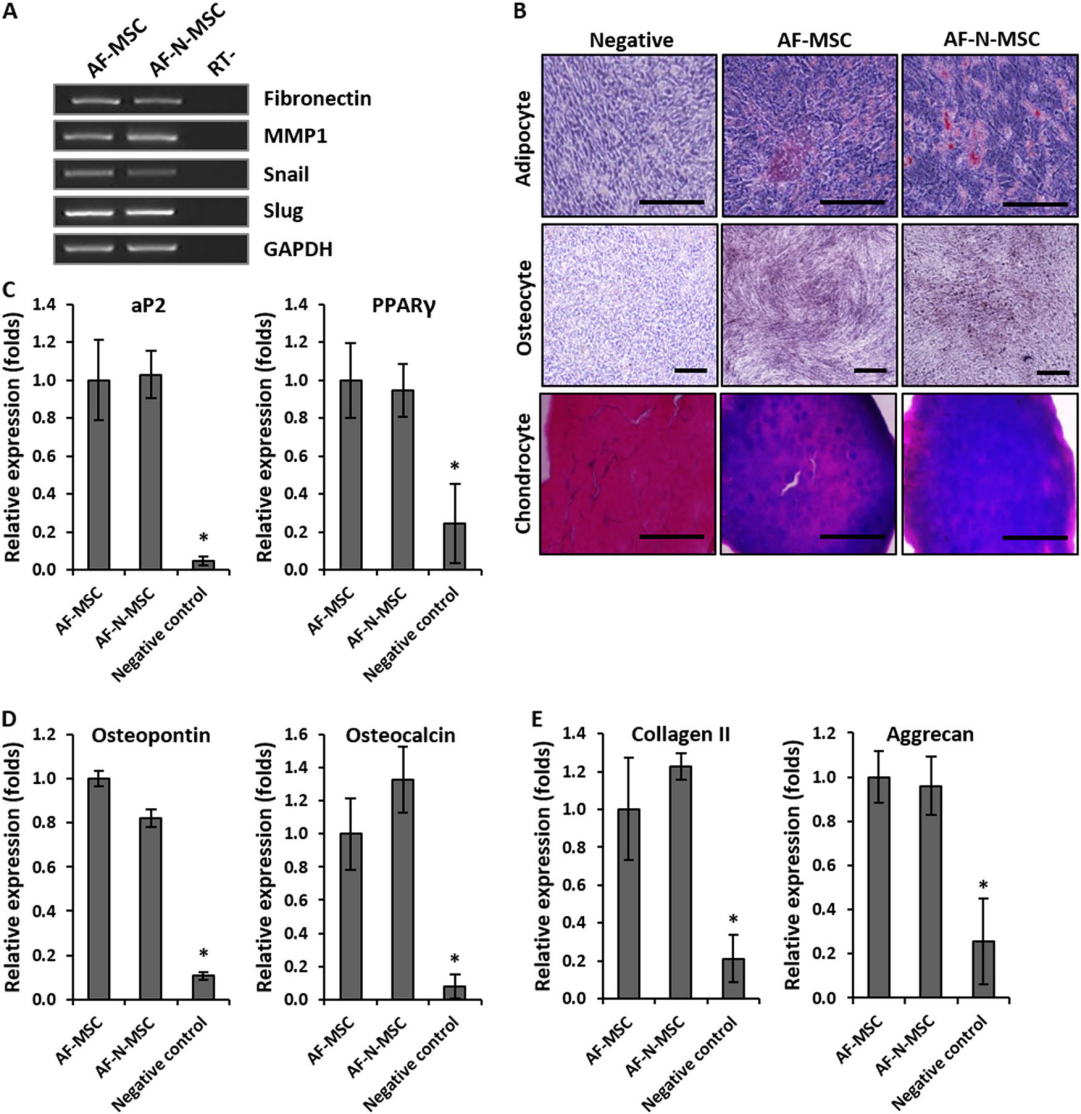
Retention of the mesenchymal stem cell (MSC) phenotype of amniotic fluid–derived MSCs (AF-MSCs). **a** Expression of mesenchymal lineage markers (fibronectin, MMP1, Snail, Slug) in AF- and AF-MSCs overexpressing Nanog (AF-N-MSCs). **b** Adipogenic, osteogenic, and chondrogenic differentiation of AF-N-MSCs at passage 20, evaluated by Oil Red O, von Kossa staining, and Alcian Blue staining, respectively. Scale bar, 200 µm. **c** mRNA expression analysis of adipogenic differentiation (aP2, PPARγ). **d** mRNA expression analysis of osteogenic differentiation (Osteopontin, Osteocalcin). **e** mRNA expression analysis of chondrogenic differentiation (Collagen II, Aggrecan). Data are represented as the mean ± SD (*n* = 3). *p < 0.05

Next, we evaluated the effect of Nanog overexpression on the identity and multipotency of MSCs by monitoring the mRNA expression of typical MSC markers, such as fibronectin, matrix metalloproteinase-1, Snail, and Slug. In addition, we assessed the effect of Nanog on adipogenic and osteogenic differentiation potential. The expression of MSC-specific markers did not differ significantly between AF- and AF-N-MSCs (Fig. 3a). Furthermore, the effect of Nanog on the multipotency of AF- and AF-N-MSCs was evaluated by their differentiation into adipocytes, osteoblasts, and chondrocytes in vitro. Figure 3b shows optical images of the adipogenic (Oil Red O staining), osteogenic (von Kossa staining), and chondrogenic (Alcian Blue) differentiation of AF-MSCs in the presence or absence of Nanog overexpression, as well as expression of markers specific to adipogenesis (aP2 and PPARγ2) (Fig. 3c), osteogenesis (osteopontin and osteocalcin) (Fig. 3d), and chondrogenesis (Collagen II and Aggrecan) (Fig. 3e) in AF-MSCs and AF-N-MSCs. These results indicate that the identity and multipotent capacity of AF-MSCs were preserved during Nanog overexpression. Thus we demonstrated that overexpression of Nanog in AF-MSCs, the pluripotency-related transcription factor selected in this study, promotes self-renewal and pluripotency-related gene expression referred to as stemness while maintaining the expression of MSC-specific markers and differentiation properties.

### Nanog-induced secretome of AF-MSCs and its paracrine effects on proliferation of DP cells

**Fig. 4:**
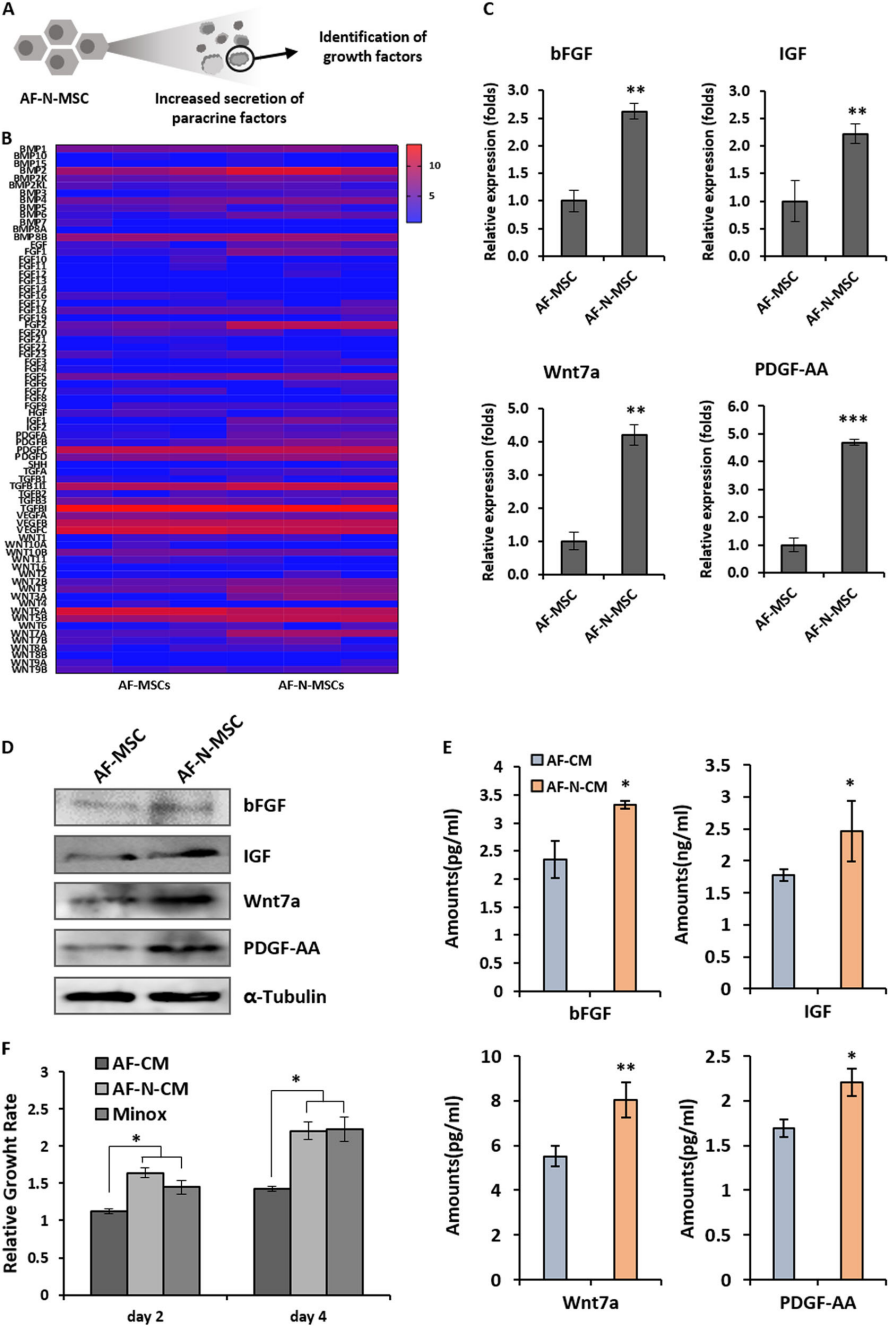
Increased expression of paracrine factors related to hair regeneration and growth in amniotic fluid–derived mesenchymal stem cells overexpressing Nanog (AF-N-MSCs). **a** Schematic describing the identification of the main paracrine factors from AF-N-MSCs. **b** Comparison of cytokine expression between AF-MSCs and AF-N-MSCs. **c** Relative mRNA expression levels of hair growth–related markers (bFGF, IGF, Wnt7a, PDGF-AA) in AF- and AF-N-MSCs. **d** Protein expression of the paracrine factors related to hair growth in AF- and AF-N-MSCs, as determined by western blot. **e** Cytokines related to hair growth secreted from AF- and AF-N-MSCs. Cytokine concentration was measured by enzyme-linked immunosorbent assays. **f** Paracrine effect of AF-N-MSC–derived conditioned media (AF-N-CM) on the growth of hair follicle dermal papilla cells. Minoxidil (1 µM) served as a positive control. Data are represented as the mean ± SD (*n* = 3). *p < 0.05; **p < 0.01; ***p < 0.001

Previous studies have demonstrated that paracrine factors secreted from MSCs promote HF induction and hair regrowth^10,22^. Therefore, we attempted to investigate whether the enhanced stemness of AF-MSCs affects the expression of paracrine factors (Fig. 4a). Before evaluating the paracrine effects of AF-N-MSCs on DP cell proliferation, we performed a microarray analysis of secretory molecules derived from three different lines of AF- and AF-N-MSCs, with the goal of characterizing Nanog-induced changes in cytokine expression (Fig. 4b). This analysis of the MSC-derived secretome provides information on paracrine-mediated therapeutic processes, as well as further elucidating the molecular and metabolic alterations underlying the roles of Nanog in AF-MSCs. The proteins upregulated in AF-N-MSCs included the bone morphogenetic protein (BMP), FGF, insulin-like growth factor (IGF), platelet-derived growth factor (PDGF), and WNT families. Among these, we focused on HF regrowth-related secretory factors, including bFGF, IGF, Wnt7a, and PDGF-AA, which were previously identified as positive regulators for hair induction^23–26^. When Nanog was overexpressed in AF-MSCs, these secretory factors were expressed at ≥2-fold higher levels in AF-N-MSCs than in AF-MSCs, and the encoded proteins were correspondingly more abundant (Fig. 4c, d). A comparison of the levels of secreted factors in AF-CM and AF-N-CM indicated that forced expression of Nanog strongly induced the secretion of paracrine factors from AF-MSCs (Fig. 4e). Finally, we assessed the effect of AF-N-CM on the proliferation of human DP cells by comparing AF-CM with medium supplemented with 1 μM minoxidil over 4 days of culture. Minoxidil, approved by Health Canada and US Food and Drug Administration, has been considered the standard medication used to promote hair regrowth for the past 20 years. This drug opens potassium channels for hyperpolarization of cell membranes, which allows more oxygen, blood, and nutrients to reach the follicle tissues. Nevertheless, topical application of minoxidil has side effects, such as irritant and allergic contact dermatitis on the scalp, and the induced hair growth disappears a few months after discontinuation of treatment^27^. The proliferation rate of HFDP cells was prominently promoted in cells treated with AF-N-CM compared to those treated with AF-CM (Fig. 4f). Here we verified the enhanced expression and secretion of paracrine factors, including HF regrowth-related secretory molecules, in AF-N-MSCs.

### In vivo effect of AF-N-CM on hair regrowth and HF cycling

**Fig. 5:**
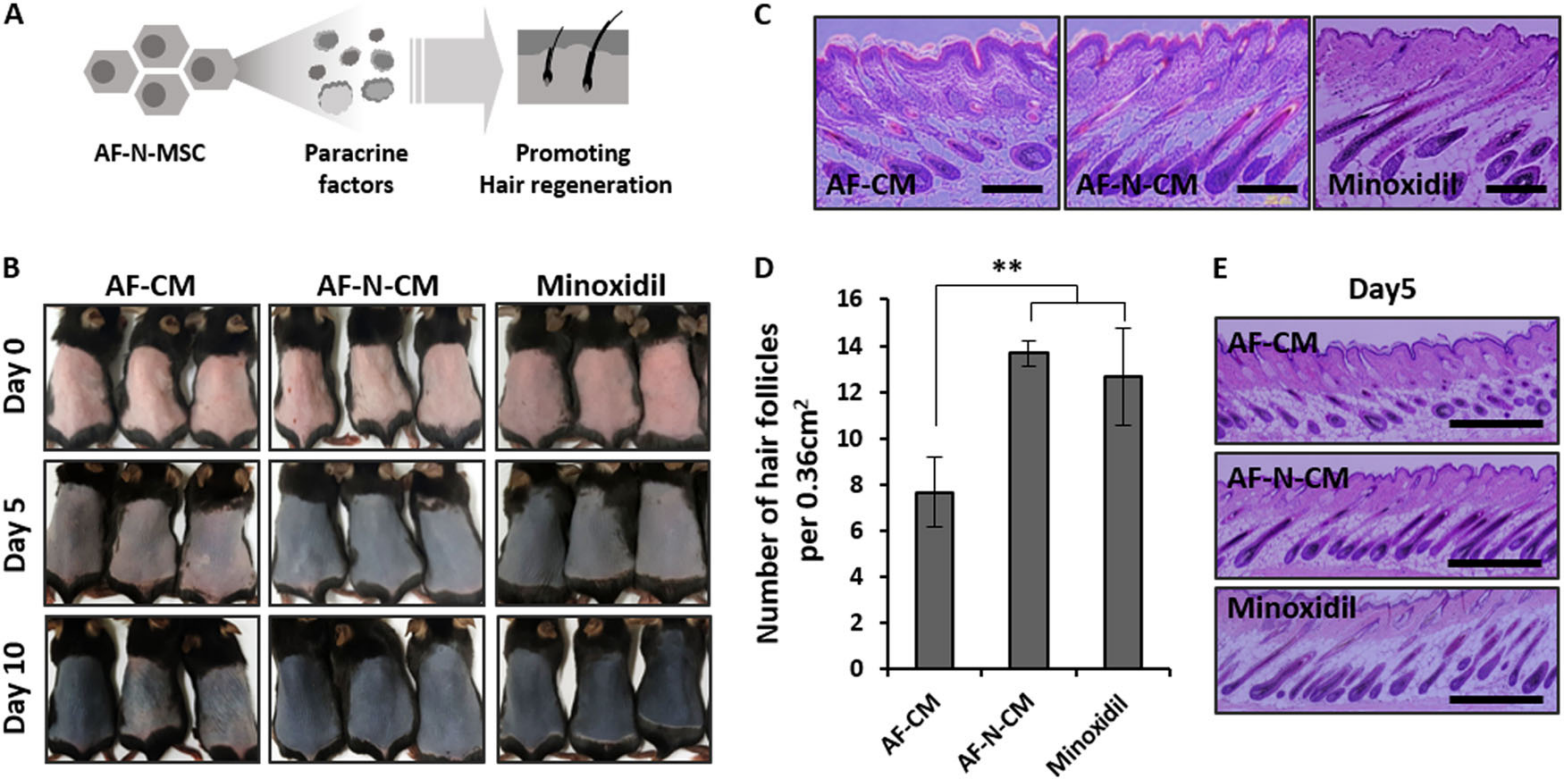
Increased hair growth in mice by amniotic fluid–derived mesenchymal stem cell overexpressing Nanog (AF-N-MSC)–derived conditioned media (AF-N-CM). **a** Schematic of a process for assessing the effect of AF-N-CM on hair regeneration. **b** Digital images of the dorsal skin of telogen C57BL/6 mice treated with AF-CM, AF-N-CM, or minoxidil. Stages of the hair cycle were determined by comparing dorsal skin colors. **c** Representative hematoxylin–eosin (H&E) images of dorsal skin sections for determination of hair follicle (HF) number and cycle. Scale bar, 200 µm. **d** HFs in 0.36 cm^2^. **e** H&E-stained dorsal skin section 5 days post-depilation. Scale bar, 600 µm. Data are represented as the mean ± SD (*n* = 3). **p < 0.01

The next question was the in vivo efficacy of AF-N-MSC-derived paracrine factors for HF regeneration (Fig. 5a). To determine whether Nanog overexpression in AF-MSCs affects the HF cycle, we harvested CM from AF- and AF-N-MSCs and topically applied 50 µL of AF-CM, AF-N-CM, or 2% minoxidil to the plucked dorsal skin of mice. Mice exposed to minoxidil served as positive controls. The stage of the hair growth cycle was determined by skin pigmentation. Regardless of the agent applied, all mice exhibited darkening of the dorsal hairs in a density-dependent manner during the initial 5-day period. The dorsal skin of AF-N-CM– and minoxidil-treated mice was fully darkened after 10 days, whereas bare spots were observed in AF-CM–treated mice (Fig. 5b). Overall, we observed distinctive differences in hair density and shaft length between the AF-CM and AF-N-CM treatments over the course of the experiment. On day 5, dorsal skin tissues were collected for histological analysis and quantification of HF density. Figure 5c, d clearly showed that AF-N-CM–treated tissues had elongated hair shafts and higher HF density than AF-CM–treated tissues. Furthermore, for all three groups, hair cycle stages could be classified based on previously reported guidelines^28^. Whereas HFs in the AF-CM–treated group were primarily in the anagen II and III stages after 5 days, those in AF-N-CM– and minoxidil-applied tissues were mainly in anagen IV and V, exhibiting narrowed DP, a hair tip that reached to hair canal, and almost complete cessation of inner root sheath development (Fig. 5e). These observations indicated that AF-N-CM promotes the entrance of secondary HFs into anagen and increases HF density to a greater extent than AF-CM.

**Fig. 6:**
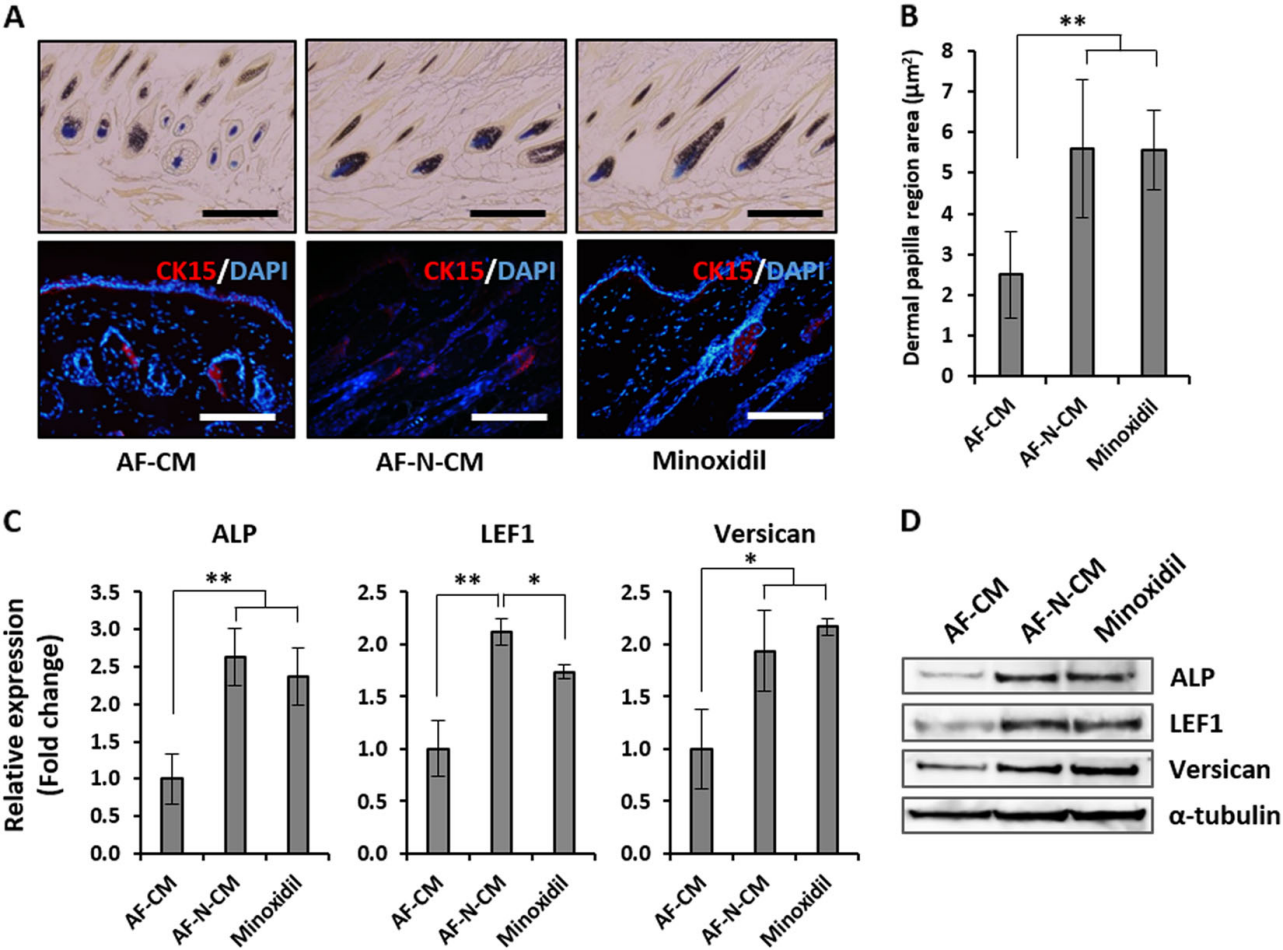
Histological analysis of amniotic fluid–derived mesenchymal stem cell overexpressing Nanog (AF-N-MSC)–derived conditioned media (AF-N-CM)-treated dorsal skin. **a** Expression patterns of the structural indicators at 5 days post-treatment: AP for hair follicle dermal papilla cell population (top) and CK15 for bulge stem cells (bottom). Quantification of the AP-positive dermal papilla region (right). Scale bar, 200 µm. **b** Quantification of the hair follicle dermal papilla region area (μm^2^) determined by ImageJ. **c** Expression of hair growth markers (ALP, LEF1, and Versican) in AF-CM, AF-N-CM, and minoxidil treatments, as determined by quantitative real-time reverse transcriptase–polymerase chain reaction. **d** Protein levels of ALP, LEF1, and Versican in AF-CM, AF-N-CM, and minoxidil treatments, as determined by western blot. Data are represented as the mean ± SD (*n* = 3). *p < 0.05, **p < 0.01

To compare the effects of AF-CM, AF-N-CM, and minoxidil on HF regeneration, we plucked dorsal hairs at the first telogen phase and, at 5 days post-application, analyzed the expression of ALP and CK15, which are well-established markers of DP cells and HF stem cells, respectively (Fig. 6a). Mice exposed to minoxidil served as positive controls. In the AF-N-CM and minoxidil groups, ALP-positive cell populations were elongated and packed, whereas AF-CM–treated DP aggregates were mostly roughly spherical. Additionally, the region of the DP population was remarkably broadened in the AF-N-CM groups compared to the AF-CM group (Fig. 6b). Meanwhile, for all three treatments, the majority of CK15 expression was detected in the bulge of anagen HFs, and in the AF-N-CM group, CK15 positivity covered a larger area of the HF bulge regions. These results indicate that the secretome of Nanog-overexpressing MSCs stimulates DP cells to accelerate HF cycling, leading to normal hair regeneration. Furthermore, we investigated the expression levels of mRNA and protein related to hair induction (ALP, LEF1, and Versican) in the dorsal skin of mice treated with AF-CM, AF-N-CM, or minoxidil (Fig. 6c). qRT-PCR analysis and western blot analysis revealed patterns similar to those observed for ALP activity, implying that paracrine factors contained in AF-N-CM can upregulate the expression of hair induction genes and accelerate hair regeneration in vivo (Fig. 6d).

## Discussion

Human MSCs have been widely regarded as a potential source of material for cell-based therapies for several reasons: self-renewal capacity, differentiation potential, immune privilege, patient-specific tissue regeneration, and circumvention of ethical restrictions. However, donor aging and cellular senescence, which decrease cell survival and diminish function, can prevent expansion of the cell mass to generate the amount of material required for successful clinical application. In this study, we attempted to surpass the limited propagation capacity via forced expression of the pluripotency-related transcription factor Nanog in human AF-MSCs. AF-N-MSCs exhibited near-linear growth during in vitro culture, whereas the proliferation capacity of non-modified AF-MSCs entered the decline stage at earlier passages (Fig. 2a). In agreement with our findings, several studies have reported that ectopic expression of pluripotency-related transcription factors, such as Oct4, Nanog, and Sox2, increases the proliferation capacity of NIH 3T3 fibroblasts and MSCs while preserving differentiation potential in cell type- and lineage-dependent manners^29,30^. Moreover, we found that overexpression of Nanog resulted in a significant extension of lifespan (37 passages in approximately 120 days of in vitro culture) relative to previous studies in which MSC cultures were supplemented with various growth factors and cytokines in an effort to promote MSC proliferation^31,32^.

Cellular senescence was first described by Hayflick (1965) in human diploid fibroblasts that underwent replicative arrest in culture^33^. Subsequently, accumulated evidence indicated that replication-induced telomere shortening activates the DNA damage response (DDR), which elicits a transient cell-cycle arrest; when the DDR becomes permanent (because the shortened telomeres cannot be repaired), it causes the cell to undergo senescence^34,35^. Oxidative stress is a major contributor to the persistent DDR during cellular senescence, whether induced by telomere shortening or direct DNA damage, and induces permanent cell-cycle arrest mediated by the p53–p21 pathway^36^. Accordingly, our results on ROS levels and p53/p21 indicate that Nanog delays passage-dependent senescence, thereby extending cellular lifespan (Fig. 2a–d). A related study showed that human first-trimester fetal human MSCs expressing Nanog and Oct4 exhibited a shortened doubling time and delayed senescence with longer telomeres than adult MSCs^37^.

We found that ectopic expression of Nanog in AF-MSCs increased endogenous expression of key pluripotency genes, such as Oct4 and Sox2 (Fig. 2e), which contradicts an earlier report that pluripotency factors are not endogenously expressed in bone marrow MSCs overexpressing Nanog^20^. Many studies have shown that Nanog expression is induced by Oct4 and Sox2, both of which bind the Nanog promoter^38^. Conversely, exogenous overexpression of Nanog increased the expression of Oct4 and Sox2 in adipose-derived stem cells and porcine nuclear-transfer embryos^39^. Although further studies are necessary to resolve the discrepancies in these results, our findings clearly showed that Nanog-mediated expression of key transcription factors is essential for the pluripotent and self-renewing phenotypes of undifferentiated MSCs. Durin*g* the in vitro expansion of MSCs, we observed no significant differences between AF- and AF-N-MSCs in the expression of MSC-specific markers or multi-differentiation potential. Our results disagree with previous reports that Nanog promotes MSC differentiation into osteoblasts but prevents adipogenesis^21,30^. To our knowledge, no other study has investigated the effects of Nanog overexpression on the differentiation of AF-MSCs into adipogenic, osteogenic, and chondrogenic lineages. Accordingly, the discrepancy between our results and those of other studies may be due to differences in MSC source, donor age, or passage number^40,41^. Overall, our findings suggest that Nanog could be a useful transcription factor to engineer high-quality MSCs by improving their stemness properties.

As a specialized mesenchymal population, DP cells play critical roles in controlling HF cycling and generating hair shafts through active communication between the DP and epithelial compartments. DP cells form a physical and chemical niche that promotes dynamic changes in HF structure and secrete diffusible factors that influence the follicular epithelium^5,42^. Furthermore, DP cell number within HFs is strongly correlated with the size of the hair shaft, a signifier of HF health. Consistent with this, when follicles are moved to sites that produce thicker types of hair, a corresponding shift in DP cell number is observed^4,43^. Conversely, miniaturization of HFs is associated with progressive hair thinning and loss and associated with failure to maintain DP cell number, and a primary defect in the DP is observed in patients with androgenetic alopecia^44^. For these reasons, improving the survival and proliferation of DP cells represents a promising strategy for managing hair loss. In this study, we found that forced expression of exogenous Nanog contributed to metabolic changes in AF-MSCs; consequently, secreted paracrine factors increased survival and proliferation of human DP cells (Fig. 4e). This effect could be attributed to the intense secretion of proteins related to hair induction, including bFGF, IGF, Wnt7a, and PDGF-AA, in AF-N-MSCs (Fig. 4b–e). These paracrine factors serve as an accelerator for activating DP cells and promoting HF regrowth^23–26^. Although uncertainty concerning the therapeutic processes by which MSCs promote tissue repair and regeneration is still widespread in the literature, the consensus mechanism includes a principal role of the paracrine factors secreted by MSCs, rather than functional recovery as a consequence of their differentiation^45^. Moreover, considering the roles of Nanog in maintaining the undifferentiated state and self-renewal of ESCs, it is not perhaps surprising that this factor can contribute to improving the stemness of MSCs, thereby revealing its superior paracrine potency and tissue repair efficacy^46^. Interestingly, based on our microarray analysis (Fig. 4b), overexpression of Nanog led to higher expression of BMPs in AF-MSCs. The role of BMP signaling in HF growth remains an open question. For example, Rendl et al. proposed a critical role of BMP signaling in which the features of DP cells are maintained and orchestrate HF formation^47^, which contradicts previous work reporting that BMPs participate in the restriction of HF cells, including bulge stem cells and matrix cells^48–50^. Accumulating evidence suggests that BMP signaling plays a regulatory role in preserving the stemness of HF stem cells and controlling HF cycling^51,52^. The present work may help elucidate certain contradictions among existing results on BMP-mediated HF growth.

Our in vivo study demonstrated that paracrine factors from Nanog-overexpressing AF-MSCs not only accelerated the transition of HFs from telogen to anagen but also significantly increased HF density and expression of hair induction genes; the magnitude of the effect was comparable to that of minoxidil treatment (Figs. 5 and 6). Although the sustained efficacy of AF-N-CM in patients with androgenic alopecia remains to be demonstrated, the elevated density of HFs observed in this study implies that its effects on hair growth are long term. In addition, we observed no adverse reaction or damage to the skin when AF-N-CM was applied, implying that a topical formulation of AF-N-CM would be nontoxic. The hair-regenerating effects of AF-N-CM on the plucked dorsal skin of mice could be attributed to the Nanog-induced increase in hair induction–related gene expression in AF-MSCs. Wnt signaling is involved in regulating the growth of HF stem cells and their progeny through their entire life cycles, including the resting (telogen) phase; thus this signaling pathway is essential for triggering and maintaining hair-inducing activity of DP cells^53^. In particular, Wnt7a, which is produced by Wnt-responsive stem cells in the bulge during telogen, promotes wound-induced HF neogenesis by mediating epidermal–mesenchymal interactions and stimulates the hair-inducing abilities of DP cells in vitro^25^. Our results showed that Nanog amplification in AF-MSCs increased the expression of autocrine Wnt7, implying that AF-N-CM treatment could contribute to the activation of anagen onset in mouse HF stem cells. Furthermore, hair cycling and regeneration are comprehensive tissue remodeling processes accompanied by growth factors, cytokines, hormones, adhesion molecules, and related enzymes. For example, Weger and Schlake demonstrated that IGF-1 affects follicular proliferation, tissue remodeling, the hair regrowth cycle, and follicular differentiation^54^. In addition, Tomita et al. showed that PDGF isoforms immediately induce entry into the anagen phase of the hair cycle at the injection site in mouse dorsal skin, leading to upregulation of signaling molecules related to HF differentiation, including Sonic hedgehog (Shh), LEF1, and Wnt5a. Conversely, mice treated with anti-PDGF antibody exhibited thinning of the dermal tissue, abnormal HF morphology, a reduction in the DP population, and thinner hair shafts relative to untreated mice^26^. In addition to IGF-1 and PDGF, bFGF localizes in the skin prior to and during HF initiation and development and plays important roles in HF morphogenesis^23^. Topical application of mouse dorsal skin with FGFs leads to earlier induction of anagen phase, a significant increase in the number and size of HFs, and prolongation of mature anagen, supported by earlier expression of β-catenin and Shh in the dermis and epidermis tissues, relative to non-treated skin^55^.

As an alternative to stem cell–based applications, the use of cell-free therapies such as MSC-derived CM would have several advantages in regenerative medicine. Specifically, AF-N-CM is (a) unencumbered by safety issues associated with transplantation of living or proliferating cells; (b) easily evaluated for dosage, efficacy, and side effects in a manner similar to conventional pharmaceutical agents; (c) more economical and practical in terms of clinical application; (d) straightforwardly mass-produced by custom-tailored cell lines; and (e) easily modified to achieve the desired cell-specific effects. This study focused on the contribution of Nanog overexpression to the secretome of AF-MSCs and the effects of AF-N-CM on hair regrowth. We found that AF-N-CM derived from Nanog-overexpressing AF-MSCs promoted proliferation of human DP cells in vitro by intense expression of hair induction proteins such as bFGF, IGF, Wnt7a, and PDGF-AA. Moreover, in vivo experiments revealed that AF-N-CM induces a profound and rapid telogen-to-anagen transition and further increases HF density, supported by upregulation of hair induction genes (ALP, LEF1, and Versican). These results suggest that, for patients experiencing hair loss, the secretome of autologous MSCs genetically engineered to overexpress Nanog could be an excellent candidate as a powerful anagen inducer and hair growth stimulator to treat alopecia.

## Supplementary information

Supplementary Table 1

Supplementary Table 2
