# Supplementary material for: Overexpression of Nanog in amniotic fluid–derived mesenchymal stem cells accelerates dermal papilla cell activity and promotes hair follicle regeneration
Source: Exp Mol Med. 2019 Jul 4;51(7):72. doi: 10.1038/s12276-019-0266-7 (PMC6802618; doi:10.1038/s12276-019-0266-7)
Supplement: Supplementary file 1 — Supplementary Table 1 [file 12276_2019_266_MOESM1_ESM.docx]

**Table 1. primer sequences for PCR**

| **Gene** | **Accesion no.** | **Primer sequence (5’-3’)** | **Anealing temperature (℃)** | **Product size (dp)** |
| --- | --- | --- | --- | --- |
| *p53* | NM_001126112.1 | F-GTTTCCGTCTGGGCTTCTTG  R-TCCGTCATGTGCTGTGACTG | 62 | 190 |
| *p21* | NM_000389.4 | F-CGACTGTGATGCGCTAATGG  R-AGCGAGGCACAAGGGTACAA | 62 | 272 |
| *Endo-Oct4* | NM_002701.5 | F-GACAGGGGGAGGGGAGGAGCTAGG  R-CTTCCCCTCCAACCAGTTGCCCCAAAC | 62 | 140 |
| *Endo-Sox2* | NM_003106.3 | F-AACCAAGACGCTCATGAAGAAG  R-GCGAGTAGGACATGCTGTAGGT | 62 | 340 |
| *Endo-Nanog* | NM_024865.4 | F-TGCAGTTCCAGCCAGCCAAATTCTCC  R-CACATTGCCAAAAGACGGCA | 62 | 340 |
| *Exo-Nanog* |  | F-GCTTGGATACACGCCGC  R-TGTTTGCCTTTGGGACTGGTGGA | 58 | 304 |
| *Fibronectin* | NM_212482.2 | F-CCCAACTGGTAACCCTTCCA  R-CTACATTCGGCGGGTATGGT | 62 | 258 |
| *MMP1* | NM_002421.2 | F-TTGAGAAAGCCTTCCAACTCTG  R-CCGCAACACGATGTAAGTTGTA | 62 | 250 |
| *Snail* | NM_005985.3 | F-ACCCCAATCGGAAGCCTAAC  R-AGCCTTTCCCACTGTCCTCA | 62 | 264 |
| *Slug* | NM_003068.4 | F-TGCGATGCCCAGTCTAGAAA  R-TTCTCCCCCGTGTGAGTTCT | 62 | 182 |
| *aP2* | NM_001442 | F-AAGAAGTAGGAGTGGGCTTTGC  R-CCACCACCAGTTTATCATCCTC | 62 | 285 |
| *PPARγ* | NM_005037 | F-TTGGTGACTTTATGGAGCCC  R-CATGTCTGTCTCCGTCTTCTTG | 62 | 311 |
| *Osteopontin* | NM_001040060 | F-GAGACCCTTCCAAGTAAGTCCA  R-GATGTCCTCGTCTGTAGCATCA | 62 | 354 |
| *Osteocalcin* | NM_199173 | F-GAGCCCCAGTTCCCCTACCC  R-GCCTCCTGAAAGCCGATGTG | 62 | 405 |
| *Collagen II* | NM_033150 | F-GAAGCTGGAAAACCAGGTGA  R-ACTTCTCCCTTCTCGCCATTAG | 62 | 392 |
| *Aggrecan* | NM_013227 | F-TCAGGAACTGAACTCAGTGG  R-GCCACTGAGTTCCACAGA | 62 | 441 |
| *bFGF* | NM_002006.5 | F-CAGATTAGCGGACGCGGTGC  R-TCACGGATGGGTGTCTCCGC | 62 | 103 |
| *IGF* | NM_000618.4 | F-CCATGTCCTCCTCGCATCTCTTCT  R-CCATACCCTGTGGGCTTGTTGAA | 62 | 163 |
| *Wnt7a* | NM_004625.3 | F-TTTCTCAGCCTGGGCATGGTCT  R-CGGCCATTGCGGAACTGAAA | 62 | 206 |
| *PDGF-AA* | NM_002607.5 | F-GCTGCAACACGAGCAGTGTCAA  R-TCCCGTGTCCTCTTCCCGATAA | 62 | 188 |
| *ALP* | NM_001631.4 | F-CCGCTTTAACCAGTGCAACA  R-CGGTTCACTGTGTGTGCGTA | 62 | 150 |
| *LEF1* | NM_016269.4 | F-CCAAACAAGGCATGTCCAGA  R-TCAGTGTGGGGATGTTCCTG | 62 | 289 |
| *Versican* | NM_004385.4 | F-GTGGGGATGAATGGAAAGGA  R-CTTGGCCTTCAGTGCTTGTG | 62 | 247 |
| *β-actin* | NM_001101.4 | F-AGCAAGCAGGAGTATGACGA  R-TGTGAACTTTGGGGGATG | 62 | 258 |
| *GAPDH* | NM_002046 | F-GTGGTCTCCTCTGACTTCAACA  R-CTCTTCCTCTTGTGCTCTTGCT | 62 | 211 |
