# Supplementary material for: Overexpression of Nanog in amniotic fluid–derived mesenchymal stem cells accelerates dermal papilla cell activity and promotes hair follicle regeneration
Source: Exp Mol Med. 2019 Jul 4;51(7):72. doi: 10.1038/s12276-019-0266-7 (PMC6802618; doi:10.1038/s12276-019-0266-7)
Supplement: Supplementary file 2 — Supplementary Table 2 [file 12276_2019_266_MOESM2_ESM.docx]

**Table 2. Antibody list for western blot and immunofluorescence**

| **Antibody** | **Company** | **Dilution** | **Host** |
| --- | --- | --- | --- |
| Oct4 | Santa Cruz Biotechnology  #sc-5279 | 1:500 | Mouse |
| Nanog | R&D systems  #AF1997 | 1:200 | Goat |
| Lin28 | Abcam  #ab46020 | 1:500 | Rabbit |
| bFGF | Abcam  #ab8880 | 1:500 | Rabbit |
| IGF | R&D systems  #MAB291 | 1:500 | Mouse |
| Wnt7A | Abcam  #ab217844 | 1:500 | Mouse |
| PDGF-AA | R&D systems  #MAB211 | 1:500 | Goat |
| CK15 | Thermo Fisher scientific  #MA5-11344 | 1:500 | Mouse |
| ALP | R&D systems  #MAB29092 | 1:500 | Mouse |
| Versican | Santa Cruz Biotechnology  #sc-25831 | 1:500 | Rabbit |
| LEF1 | Santa Cruz Biotechnology  #sc-374412 | 1:500 | Mouse |
| Cy3 Anti-  Goat IgG | Jackson ImmunoResearch Labs #715–165-151 | 1:500 | Donkey |
| Cy3 Anti-Mouse IgG | Jackson ImmunoResearch Labs #705–165-147 | 1:500 | Donkey |
| Tubulin | Sigma Aldrich  #T5168 | 1:1000 | Mouse |
